# Molecular Docking Integrated with Network Pharmacology Explores the Therapeutic Mechanism of *Cannabis sativa* against Type 2 Diabetes

**DOI:** 10.3390/cimb45090457

**Published:** 2023-09-01

**Authors:** Juan Manuel Guzmán-Flores, Victoriano Pérez-Vázquez, Fernando Martínez-Esquivias, Mario Alberto Isiordia-Espinoza, Juan Manuel Viveros-Paredes

**Affiliations:** 1 Instituto de Investigación en Biociencias, Centro Universitario de los Altos, Universidad de Guadalajara, Tepatitlán de Morelos C.P. 47620, Jalisco, Mexico; fernando.mesquivias@academicos.udg.mx; 2 Department of Medical Sciences, University of Guanajuato, Campus León, León C.P. 37220, Guanajuato, Mexico; vicpe@yahoo.com; 3 Instituto de Investigación en Ciencias Médicas, Departamento de Clínicas, Centro Universitario de los Altos, Universidad de Guadalajara, Tepatitlan de Morelos C.P. 47620, Jalisco, Mexico; mario.isiordia@academicos.udg.mx; 4 Laboratorio de Investigación y Desarrollo Farmacéutico, Departamento de Farmacobiología, Centro Universitario de Ciencias Exactas e Ingenierías, Universidad de Guadalajara, Guadalajara C.P. 44430, Jalisco, Mexico; juan.viveros@academicos.udg.mx

**Keywords:** diabetes, *Cannabis sativa*, network pharmacology, molecular docking, gene ontology

## Abstract

The incidence of type 2 diabetes (T2D) is rising, and finding new treatments is important. *C. sativa* is a plant suggested as a potential treatment for T2D, but how it works needs to be clarified. This study explored the pharmacological mechanism of *C. sativa* in treating T2D. We identified the active compounds in *C. sativa* and their targets. From there, we examined the genes associated with T2D and found overlapping genes. We conducted an enrichment analysis and created a protein–protein and target–compound interactions network. We confirmed the binding activities of the hub proteins and compounds with molecular docking. We identified thirteen active compounds from *C. sativa*, which have 150 therapeutic targets in T2D. The enrichment analysis showed that these proteins are involved in the hormone, lipid, and stress responses. They bind transcription factors and metals and participate in the insulin, PI3K/Akt, HIF-1, and FoxO signaling pathways. We found four hub proteins (EGFR, ESR1, HSP90AA1, and SRC) that bind to the thirteen bioactive compounds. This was verified using molecular docking. Our findings suggest that *C. sativa*’s antidiabetic action is carried out through the insulin signaling pathway, with the participation of HIF-1 and FoxO.

## 1. Introduction

Type 2 diabetes (T2D) is a complex, multifactorial disease that affects various body organs and causes changes in carbohydrate, lipid, and protein metabolism [1]. It is characterized by elevated blood glucose levels. In the last 40 years, there has been a significant increase in the incidence of this disease, both in men and women [2].

The etiology of T2D includes insulin resistance, alterations in the secretion of this hormone, and alterations in the immune, gastrointestinal, and nervous systems [1]. The treatment for T2D should be comprehensive and include diet modifications, exercise, and drugs that enhance insulin secretion and overcome insulin resistance or various drugs, such as peroxisome-proliferator receptor agonists (PPARs), glucagon-receptor agonists, glucokinase activators, and 11 β-hydroxysteroid dehydrogenase inhibitors [3,4]. In addition, new therapeutic options, such as different types of nanoparticles synthesized from selenium, gold, zinc, silver, and plants from different world regions, have also been tested in recent years [5,6,7,8].

*C. sativa* is a plant native to central Asia, used for recreational, religious, and medicinal purposes. This plant contains a large number of compounds, such as cannabinoid phenols, non-cannabinoid phenols, flavonoids, terpenoids, alcohols, aldehydes, n-alkanes, wax esters, steroids, and alkaloids [9,10]. Traditionally, its consumption has been associated with negative behaviors. It has also been reported that excessive consumption can lead to alterations in the central nervous system, adverse effects on the respiratory and cardiovascular systems, and aggravate psychiatric conditions [11].

Nevertheless, *C. Sativa* has also been used to treat pain, nausea, and vomiting in patients with various diseases, such as rheumatism, epilepsy, and asthma [10,11]. *C. Sativa* has also been used as a treatment for T2D, but the results have been controversial. One study reported that the recreational use of *C. sativa* may increase the risk of peripheral arterial occlusion, myocardial infarction, and renal disease in patients with T2D [12]. On the other hand, a meta-analysis reported an inverse association between diabetes and cannabis smoking [13], yet another study corroborated the latter finding, although only in females, not males [14]. However, the mechanism by which *C. sativa* acts, considering the compounds it contains, has not been fully elucidated.

Therefore, this study aimed to explore the components, targets, and pathways of *C. sativa* in T2D using network pharmacology and molecular docking to provide a reference for future drug development for treating T2D.

## 2. Materials and Methods

### 2.1. Screening for Potential Active Compounds in C. sativa

We used the Traditional Chinese Medicine Systems Pharmacology Database and Analysis Platform (TCMSP, https://tcmsp-e.com/tcmsp.php, accessed on 5 March 2023) [15] and entered the word *C. sativa* to obtain the corresponding compounds and their related information. According to the absorption, distribution, metabolism, and excretion (ADME) protocols, the active compounds were screened and the criteria were an oral bioavailability (OB) of ≥20 and drug-likeness (DL) of ≥0.10, according to the criteria suggested by the same database and supported by two previous studies [16,17].

### 2.2. Searching for Potential Target Genes for Bioactive Compounds from C. sativa

Putative targets of the selected compounds of *C. sativa* were predicted using SwissTarget Prediction (http://www.swisstargetprediction.ch/, accessed on 15 March 2023) [18] and PharmMapper (http://www.lilab-ecust.cn/pharmmapper/, accessed on 15 March 2023) [19]. The SDF or SMILES formats of the structures of these molecular bioactive compounds were obtained from the PubChem database (https://pubchem.ncbi.nlm.nih.gov/, accessed on 15 March 2023) [20] and uploaded to the servers. In the SwissTarget Prediction platform, we filtered the results by the species Homo Sapiens and only targets with a probability of ≥0.1 were considered. The parameters used in PharmMapper were Maximum Generated Conformations 300, Human Protein Targets Only, and hit target pharmacophore models, which were listed by normalized fit score, discarding those with scores less than 0.5. Finally, the UniProt database (https://www.uniprot.org/, accessed on 15 March 2023) [21] was used to obtain the unique corresponding gene names and UniProt IDs.

### 2.3. Mining for Genes Related to T2D

The T2D-related genes were obtained by retrieving MalaCards (https://www.malacards.org/, accessed on 20 March 2023) [22], the DisGeNet database (https://www.disgenet.org/, accessed on 20 March 2023) [23], and the Comparative Toxicogenomics Database (CTD) (http://ctdbase.org/, accessed on 20 March 2023) [24]. The MalaCards disease and disorders database is organized into “disease cards”. DisGeNET is a discovery platform that contains one of the largest public collections of genes and variants related to human diseases; targets with a score of ≥0.1 were screened. CTD is a robust, publicly available database that aims to advance the understanding of how environmental exposure affects human health; only curated targets were selected from this database.

Finally, we created a Venn diagram to show the intersection between the T2D-related genes and the predicted targets of the bioactive compounds from *C. sativa*. To construct and visualize the Venn diagram, we employed the Venny 2.1 platform (https://bioinfogp.cnb.csic.es/tools/venny/, accessed on 20 March 2023).

### 2.4. Construction and Analysis of the Protein–Protein Interaction Network

We constructed the protein–protein interaction network using the STRING database (https://string-db.org/, accessed on 25 March 2023) [25], limiting the network to the Homo Sapiens species and a confidence score of >0.9. The network was then imported into the Cytoscape software, v3.9.1 [26], a freely available graphical user interface for importing, visually exploring, and analyzing biomolecular interaction networks. Nodes represented the network’s active constituents and target genes, while edges indicated interactions between the active compounds and their target genes. We analyzed the network with the CytoHubba plug-in [27], which provides a user-friendly interface for exploring the important nodes in biological networks.

### 2.5. Pathway and Functional Enrichment Analysis

The intersecting genes were evaluated via Gene Ontology (GO) and Kyoto Encyclopedia of Genes and Genomes (KEGG) enrichment analyses using the Shiny GO 0.77 tool (http://bioinformatics.sdstate.edu/go/, accessed on 27 March 2023) [28], with an FDR of <0.05 and *p* of <0.05 as cut-off values, displaying the top ten results.

### 2.6. Construction of the Target–Bioactive-Compound Network of C. sativa

To explain the relationship between the compounds and targets, a herb–compound–target network was built and visualized using Cytoscape.

### 2.7. Molecular Docking

Finally, we made an intersection between the hub proteins of the protein–protein interaction network and the proteins of the target–bioactive-compound network; the selected proteins were docked with their respective bioactive compounds. Molecular docking was performed with the Swiss dock database (http://www.swissdock.ch/) [29] using the predefined parameters. The docking type was blind, and we chose the accurate option on the website and allowed a flexibility of 0 A° for the side chains of any ligand atom. The atom charges and hydrogen atoms were added to the protein, and the solvents were removed with the UCSF Chimera. The target proteins were obtained from the Protein Data Bank (https://www.rcsb.org/) [30] or AlphaFold Protein Structure Database (https://alphafold.ebi.ac.uk/) [31]. For the molecular docking of the SRC protein, we used the structure 1A07; for the EGFR, ESR1, and HSP90AA1 proteins, we used the structures obtained from Alphafold (I3WA68, B0QYW7, and A0A0U1RR69, respectively). The chemical structures of the active components were downloaded from the PubChem database, and the Openbabel software, v2.4.0 [32] was used to convert the SDF format into the mol2 format. Then, the results of the interactions were visualized in Chimera UCSF and BIOVIA Discovery Studio (https://discover.3ds.com/discovery-studio-visualizer-download, accessed on 25 April 2023) [33]. Lower scores represent a more stable binding affinity of protein and ligand.

## 3. Results

### 3.1. Screening of Active Compounds and Targets in C. Sativa

Through the Traditional Chinese Medicine Systems Pharmacology Database and Analysis Platform (TCMSP), we found 47 compounds from the *C. sativa* plant. However, after filtering the results with the parameters of an OB of ≥20 and DL of ≥0.10, there were 13 remaining compounds, including Apigenin, Arachidonic acid, Cannabinol, Caryophyllene oxide, Gamma-Linolenic acid, Gondoic acid, Linoleic Acid, Linolenic Acid, Luteolin, n-cis-Feruloyltyramine, Oleic Acid, Sitosterol, and Stigmasterol. Interestingly, both cannabidiol and tetrahydrocannabinol showed a low OB (3.97 and 13.65, respectively), for which reason they were not considered in this investigation; however, it is to be expected that, through other routes of administration, these molecules may have pharmacological effects. Table 1 shows the thirteen final bioactive compounds and some of their characteristics.

We used PharmMapper and SwissTarget Prediction to identify the targets corresponding to the bioactive compounds of *C. sativa*. In general, we found more targets on the PharmMapper platform, but the vast majority of targets matched on both platforms used. Table 1 shows the total targets for each bioactive compound after eliminating the repeated targets, and the total number of targets of all the active compounds was 509. This table shows that the three compounds with the highest number of targets were Gamma-Linolenic acid, Linoleic Acid, and Linolenic Acid.

### 3.2. Exploration of the Possible Therapeutic Targets of C. sativa in Treating T2D

We obtained 1300 genes associated with T2D from the MalaCards, DisGeNet, and Comparative Toxicogenomics Databases once the duplicates had been removed. Specifically, we found 590 genes in the MalaCards database, 958 genes in the DisGeNet database, and 234 genes in the Comparative Toxicogenomics Database (CTD).

**Figure 1:**
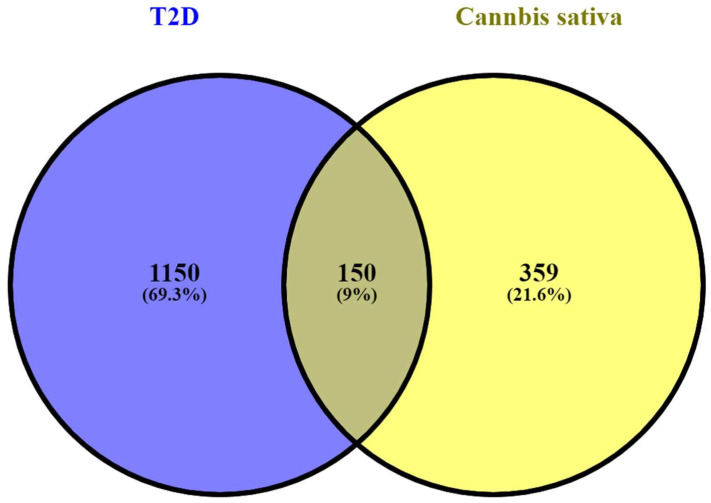
Venn diagram of *C. sativa*-related targets and T2D-related genes. One hundred fifty common genes were found between T2D and *C. sativa* targets.

Subsequently, common T2D genes and plant-related targets were obtained using a Venn diagram. A total of 150 potential anti-T2D genes from *C. sativa* were selected and considered as key targets. This diagram is shown in Figure 1.

### 3.3. Enrichment Analysis of Overlapping Targets

**Figure 2:**
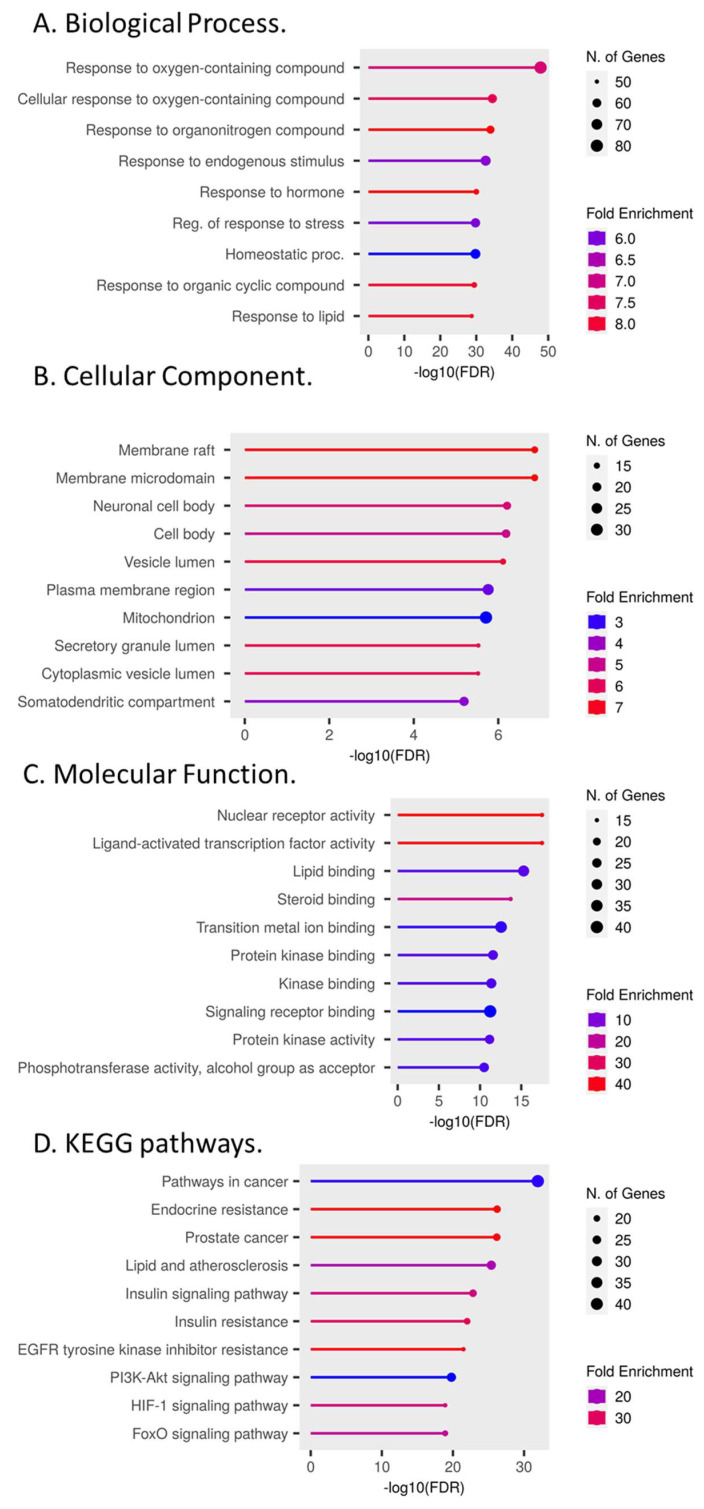
Representation of functional annotation and enriched pathways. (**A**) GO in terms of biological processes. (**B**) GO in terms of cellular components. (**C**) GO in terms of molecular function. (**D**) KEGG pathway analysis.

In the Gene Ontology (GO) enrichment, we showed the top 10 of the 150 overlapping targets of each enrichment. According to the results of our Biological Process (BP) (Figure 2A), the function of the bioactive compounds mainly focused on the response to oxygen- and nitrogen-containing compounds and the response to hormones, lipids, and stress. Most genes coded for proteins in the cell membrane, vesicles, and mitochondria (Figure 2B). The Molecular Function (MF) items (Figure 2C) mainly included binding to transcription factors, lipids, metals, and protein kinases. The Kyoto Encyclopedia of Genes and Genomes (KEGG) pathway enrichment analysis showed that *C. sativa* was mainly involved in cancer-related pathways, lipids, and insulin signaling (Figure 2D).

### 3.4. Protein–Protein Interaction Network Analysis

**Figure 3:**
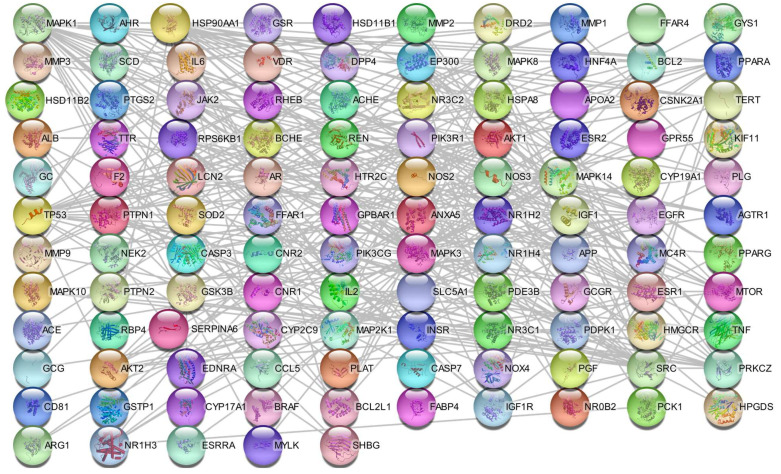
Protein–protein interaction network of overlapping genes between *C. sativa* targets and T2D-associated genes.

The Protein–Protein interaction (PPI) network was constructed with the Search Tool for the Retrieval of Interacting Genes/Proteins (STRING) database and imported into Cytoscape (Figure 3). In this network, nodes represent proteins and edges represent protein–protein interactions. The proteins with the most interactions were Serine/threonine-protein kinase (AKT1), MAP kinase-activated protein kinase 3 (MAPK3), Mitogen-activated protein kinase 1 (MAPK1), Proto-oncogene tyrosine-protein kinase Src (SRC), Heat shock protein HSP 90-alpha (HSP90AA1), TP53-binding protein 1 (TP53), Estrogen receptor (ESR1), Phosphatidylinositol 4,5-bisphosphate 3-kinase catalytic subunit alpha isoform (PIK3R1), Histone acetyltransferase p300 (EP300), and Epidermal growth factor receptor (EGFR), with 29, 26, 26, 25, 25, 24, 22, 21, 20, and 17 interactions of each molecule, respectively.

### 3.5. Exploration of the Possible Therapeutic Targets of C. sativa in Treating T2D

**Figure 4:**
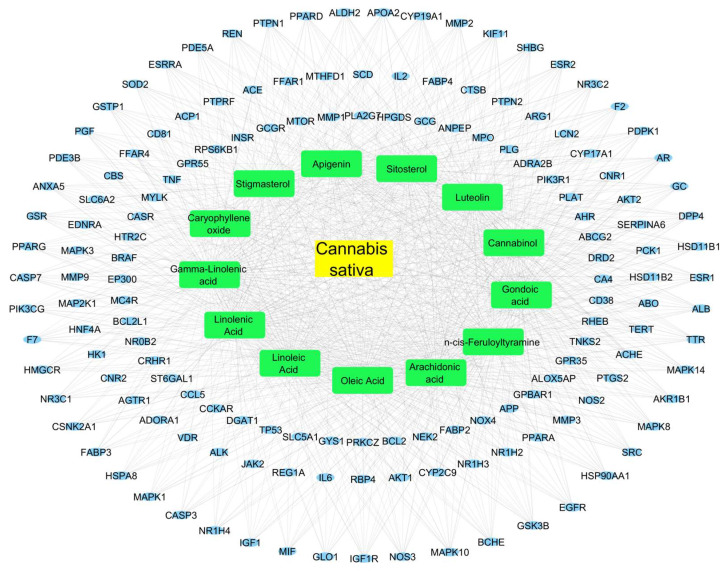
*C. sativa* target–compound network in the treatment of T2D. In the center, in yellow rectangles, is *C. sativa*; then, in green rectangles are the plant’s bioactive compounds; at the end, in blue circles, are the genes associated with T2D and the targets of the bioactive compounds.

We constructed a compound–target network from the 150 common genes between the T2D and C. *sativa* targets. This network can be seen in Figure 4. From the analysis of this network, we identified 24 targets that bound to the 13 bioactive compounds of *C. sativa*. These targets were Aldo-keto reductase family 1 member B1 (AKR1B1), Albumin (ALB), Androgen receptor (AR), Cholinesterase (BCHE), Dipeptidyl peptidase 4 (DPP4), EGFR, ESR1, Estrogen receptor beta (ESR2), Prothrombin (F2), Vitamin D-binding protein (GC), Glycogen synthase kinase-3 beta (GSK3B), 11-beta-hydroxysteroid dehydrogenase 1 (HSD11B1), HSP90AA1, Insulin-like growth factor 1 receptor (IGF1R), Kinesin-like protein (KIF11), Mitogen-activated protein kinase 10 (MAPK10), MAPK14, MAPK8, Nitric oxide synthase, endothelial (NOS3), Mineralocorticoid receptor (NR3C2), 3-phosphoinositide-dependent protein kinase 1 (PDPK1), Sex hormone-binding globulin (SHBG), SRC, and Transthyretin (TTR).

We then analyzed the intersection between the hub proteins of the protein–protein interaction network and the 24 targets that bound the 13 bioactive compounds. We found four common targets, EGFR, ESR1, HSP90AA1, and SRC; therefore, these targets were considered to be the most important. These four targets were then verified using a molecular docking analysis.

### 3.6. Molecular Docking of Key Targets

**Figure 5:**
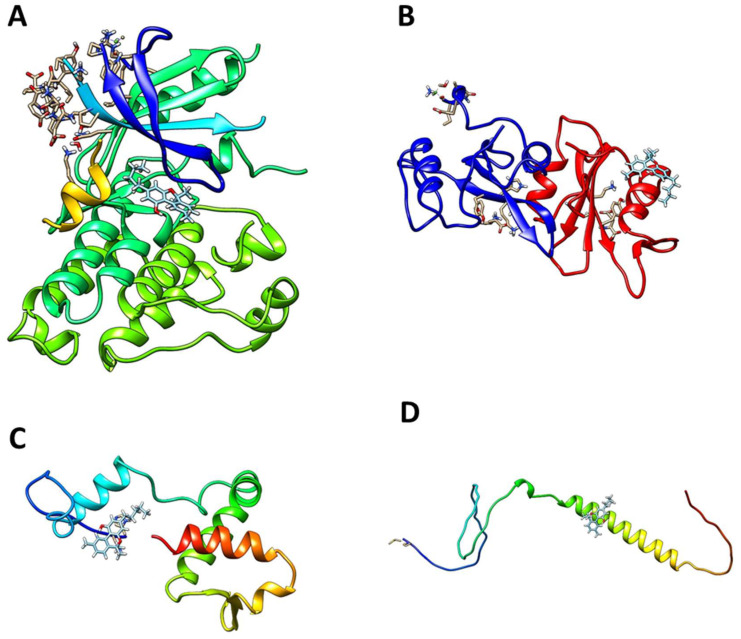
Molecular docking results: (**A**) cannabinol to EGFR; (**B**) cannabinol to SRC; (**C**) cannabinol to ESR1; and (**D**) cannabinol to HSP90AA1.

**Figure 6:**
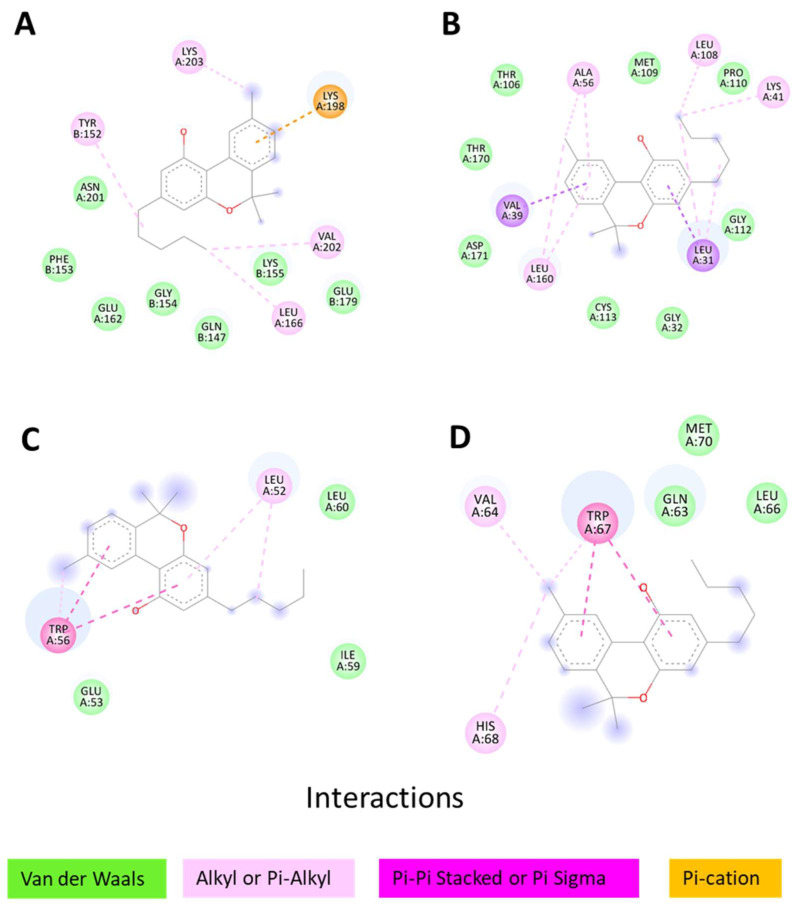
Types of bonds between proteins and cannabinol. (**A**) cannabinol to EGFR; (**B**) cannabinol to SRC; (**C**) cannabinol to ESR1; and (**D**) cannabinol to HSP90AA1.

According to the results of the intersection between the top ten hub proteins and the targets with the highest number of interactions, we carried out the molecular docking of the target proteins and active compounds involved. The binding energy between the target proteins and active compounds was approximately between −5.78 and −9.09 kcal mol^−1^ (Table 2). The results showed that the ∆G of the four proteins bound to the thirteen bioactive computations of *C. sativa* was less than 0, indicating that they all spontaneously bound to each other. Finally, we chose, as an example, the docking performed between cannabinol and the four target proteins for visualization (Figure 5). Figure 6 shows the different types of bonds between the proteins and cannabinol.

## 4. Discussion

T2D is a multifactorial and complex disease affecting many of the world’s adult population. The treatment for this disease consists mainly of a series of measures, including diet, exercise, and antihyperglycemic drugs. However, despite all these therapeutic measures, the side effects of T2D still cannot be adequately controlled. Therefore, searching for new therapeutic targets and drugs against this disease is vital for the health sector.

This work explored the components, targets, and effects produced by *C. sativa* used in T2D. *C. sativa* possesses thirteen compounds with possible pharmacological activity, each with many targets. Moreover, our research showed that 1300 genes are associated with T2D and 150 overlap with *C. sativa* targets. The overlapping genes involved hormone response, stress, cancer pathways, and insulin resistance. In addition, four targets are among the top ten proteins with the highest degree and bind with the thirteen *C. sativa* compounds, which was further verified using a molecular docking analysis.

According to the principle of absorption, distribution, metabolism, and excretion (ADME) protocols (setting OB ≥ 20 and DL ≥ 0.10) and the number of targets, we found that Linoleic Acid, Linolenic Acid, and Gamma-Linolenic acid were the compounds that had the highest number of targets within the ADME parameters. Therefore, these three compounds could be thought to have the highest pharmacological activity in T2D. However, it should be considered that these compounds are not exclusive to *C. sativa*, and the idea is that the pharmacological action of plants results from the activity of their numerous compounds and not only some of them.

Both Linoleic Acid and Linolenic Acid have been widely and inversely associated with T2D. Generally, it has been reported that a high intake of these fatty acids improves insulin resistance, reduces the risk of T2D, and always takes care of an adequate ratio between these two types of fatty acids. Specifically, omega-3 fatty acids, such as Linolenic acid, can suppress the expression of genes related to inflammation interacting with peroxisome proliferator-activated receptors, hepatocyte nuclear factor- 4a, and liver X receptor, or through mitigating the activation of the NF-kB transcription factor [34,35,36,37]. In some cases, this effect is only observed in the presence of a genetic factor; it has been reported that, in subjects with a high genetic risk for T2D, the modulatory effect of linolenic acid is suppressed [37]. In T2D, lipid metabolism is altered. A previous study reported that mice treated with these fatty acids had a significantly reduced liver weight, hepatic cholesterol levels, and cholesterol synthesis enzyme farnesyl pyrophosphate synthase expression. Specifically, Linolenic Acid increases the expression of acetyl-CoA oxidase-associated proteins and suppresses PPARα-induced proteins. Linoleic Acid decreases the hepatic expression of fatty-acid-binding protein (FABP)-1/FABP4 levels [38].

Moreover, Gamma-Linolenic acid also seems to act on glucose metabolism [39]. Specifically, in a murine model of T2D, Gamma-Linolenic acid reduced the serum lipid and glucose levels, as well as the proteins that regulate adipocyte function. The decrease in glucose may have been because this fatty acid modifies insulin levels and sensitivity and increases insulin-dependent glucose utilization through oxidation and conversion to fatty acids [40]. Similarly, most of the other active compounds of *C. sativa* found in this study have been associated with T2D [41,42,43,44,45,46,47].

Regarding the results of our gene ontology enrichment, the Biological Processes enriched by *C. sativa* in T2D corresponded to the response to hormones, stress, and lipids, among others. Therefore, these results may indicate that *C. sativa* can regulate the hormones involved in the pathophysiology of T2D, such as insulin [48]. We also found the Cellular Component of the mitochondria to be enriched, indicating that the targets of *C. sativa* are found in this organelle. Among the multiple alterations of T2D is mitochondrial dysfunction, leading to high levels of reactive oxygen species and low levels of ATP [49]. In other studies, it has been reported that drugs against T2D affect the oxidative phosphorylation in mitochondria; even therapeutic targets present in this organelle have been proposed [50]. Therefore, we can assume that the bioactive compounds from *C. sativa* affect the mitochondria in patients with T2D. The main Molecular Functions enriched were those related to lipid binding and protein kinases. Protein kinases are a group of molecules that regulate a wide variety of cellular processes through protein phosphorylation and have been implicated in the pathophysiology of T2D. They have even been proposed as therapeutic targets [51]. However, protein kinases have the disadvantage of a low specificity. However, it is interesting that *C. sativa* also affects the activity of these proteins.

We found that the insulin signaling pathway, PI3K/Akt signaling pathway, Hypoxia-inducible factor 1 (HIF-1) signaling pathway, FoxO signaling pathway, and cancer-related pathways such as prostate cancer were the potential fundamental mechanisms of *C. sativa* in the treatment of T2D. Interestingly, the insulin signaling pathway is regulated by *C. sativa* in T2D, as this would largely explain the protective effect of this plant for this disease, as previously reported in epidemiological studies [13,14]. The PI3K/Akt signaling pathway is closely related to the insulin signaling pathway, which are both altered in T2D [52]. Other research has reported that another *C. sativa* compound, delta-nine tetrahydrocannabinol, can regulate this metabolic pathway, which supports our results [53]. The PI3K/Akt pathway regulates the HIF-1 signaling pathway, and it has been widely reported that hypoxia and inflammation can lead to obesity-induced insulin resistance. However, it has also been reported that the genetic or pharmacological inhibition of HIF-1 can prevent or reverse this insulin resistance. Thus, *C. sativa* may also be inhibiting HIF-1 [54]. Another metabolic pathway enriched by the action of C. in T2D is the FoxO signaling pathway, which is also regulated by the PI3K/Akt pathway. Since FoxO can regulate glucose metabolism through gluconeogenesis, it is expected that *C. sativa* also regulates glucose through this metabolic pathway.

From the analysis of the hub proteins of the protein–protein interaction network and the target–compound network of *C. sativa*, we found four key therapeutic targets (EGFR, ESR1, HSP90AA1, and SRC) for the thirteen active compounds of *C. sativa*. These results were subsequently verified through molecular docking. As expected, according to the structure of cannabinol, most of the interactions were alkyl, pi-alkyl, and Van der Waals forces.

EGFR and ESR1 are molecules mainly involved in cancer. However, it has also been shown that the PI3K/Akt pathway can be activated through these receptors. ESR1 induces Solute carrier family 2, facilitated by glucose transporter member 4 (GLUT4) translocation, and thus potentiates glycolysis [55,56]. Therefore, we can assume that the bioactive compounds of *C. sativa* bind to EGFR and ESR1, activate the PI3K/Akt pathway, and induce glycolysis, thus decreasing serum glucose levels. Similarly, HSP90AA1 and SRC are related to the PI3K/Akt pathway [57,58]. In addition, a pharmacological network analysis identified both proteins as therapeutic targets of different plants in T2D [59,60,61].

Therefore, this research provides valuable information on the mechanisms of action of thirteen different bioactive compounds from *C. sativa* on T2D. However, it also has some limitations, mainly because these results need to be experimentally verified. Another point to consider is that the TCMSP database only contains 47 compounds of *C. sativa*, when more than 500 plant compounds have been described. Another fact to think about regarding molecular docking is that this type of analysis only considers the interaction between the two molecules to be studied, when, in reality, many other molecules can compete for the same binding site; in addition, they are continuously in motion. The dosage of these compounds must also be considered to observe a pharmacological action. Finally, it is also possible that pharmacological interactions occur with drugs are approved to treat T2D, since it must be regarded that the administration of herbs does not replace the treatments accepted by medical associations, but only complements them.

## 5. Conclusions

After conducting our research, molecular docking in combination with network pharmacology contributed to a better understanding of the therapeutic mechanism of C. sativa in T2D. We pinpointed thirteen compounds with pharmacological activity in *C. sativa*, a plant with 509 human therapeutic targets. In our search for genes associated with T2D, we came across 1300 genes linked to this disease. By comparing these two sets of results, we found 150 genes that overlapped. We inferred from our analysis of these genes that their products involve cell membrane and mitochondria functions and hormone, lipid, and stress responses. Furthermore, we discovered that *C. sativa*’s antidiabetic properties are carried out through the insulin signaling pathway, specifically the PI3K/Akt pathway, in which HIF-1 and FoxO also play a role. Additionally, we identified four essential proteins (EGFR, ESR1, HSP90AA1, and SRC) as therapeutic targets of *C. sativa*, all of which are linked to the PI3K/Akt pathway.

## Figures and Tables

**Table 1:** Active compounds of *C. sativa*, their properties, structures, and number of targets.

Molecule ID	Molecule Name	MW	OB (%)	DL	PubChem CID	Targets
MOL000008	Apigenin	270.25	23.06	0.21	5280443	64
MOL001439	Arachidonic acid	304.52	45.57	0.20	444899	88
MOL005028	Cannabinol	310.47	22.04	0.32	2543	76
MOL002003	Caryophyllene oxide	220.39	32.67	0.13	1742210	34
MOL002683	Gamma-Linolenic acid	278.48	45.01	0.15	5280933	93
MOL005030	Gondoic acid	310.58	30.70	0.20	5282768	81
MOL000131	Linoleic Acid	280.50	41.90	0.14	5280450	90
MOL000432	Linolenic Acid	278.48	45.01	0.15	5280934	92
MOL000006	Luteolin	286.25	36.16	0.25	5280445	73
MOL000483	n-cis-Feruloyltyramine	313.38	55.00	0.26	6440659	85
MOL000675	Oleic Acid	282.52	33.13	0.14	445639	89
MOL000359	Sitosterol	414.79	36.91	0.75	12303645	66
MOL000449	Stigmasterol	412.77	43.83	0.76	5280794	64

MW, Molecular Weight; OB, Oral Bioavailability; DL, Drug-Likeness; and PubChem CID, PubChem’s compound identifier. The chemical structures of the compounds studied are shown in the Supplementary Material.

**Table 2:** The binding energy of potentially active compounds from *C. sativa* and their four target proteins.

Compounds	EGFR (kcal/mol)	SRC (kcal/mol)	ESR1 (kcal/mol)	HSP90AA1 (kcal/mol)
Apigenin	−7.55	−6.88	−6.56	−6.69
Arachidonic acid	−9.09	−6.08	−7.24	−6.78
Cannabinol	−7.44	−6.38	−6.77	−6.47
Caryophyllene oxide	−6.85	−5.78	−6.15	−6.16
Gamma-Linolenic acid	−8.85	−6.24	−6.83	−6.47
Gondoic acid	−9.07	−6.62	−7.40	−6.80
Linoleic Acid	−8.74	−6.46	−7.08	−6.60
Linolenic Acid	−8.56	−6.82	−7.31	−6.19
Luteolin	−7.37	−6.99	−6.70	−6.19
n-cis-Feruloyltyramine	−7.70	−6.64	−7.13	−6.33
Oleic Acid	−7.98	−6.52	−7.29	−6.88
Sitosterol	−7.60	−6.76	−7.16	−6.27
Stigmasterol	−7.64	−6.11	−7.29	−6.70

## Data Availability

Data are contained within the article.

## Supplementary Materials

The following supporting information can be downloaded at: https://www.mdpi.com/article/10.3390/cimb45090457/s1, Figure S1: Chemical structures of the studied compounds of *C. sativa*.
