# Supplementary material for: Molecular Docking Integrated with Network Pharmacology Explores the Therapeutic Mechanism of Cannabis sativa against Type 2 Diabetes
Source: Curr Issues Mol Biol. 2023 Sep 1;45(9):7228–41. doi: 10.3390/cimb45090457 (PMC10529732; doi:10.3390/cimb45090457)
Supplement: Supplementary file 1 [file cimb-45-00457-s001.zip › cimb-2575946-supplementary.pptx]

## Slide 1
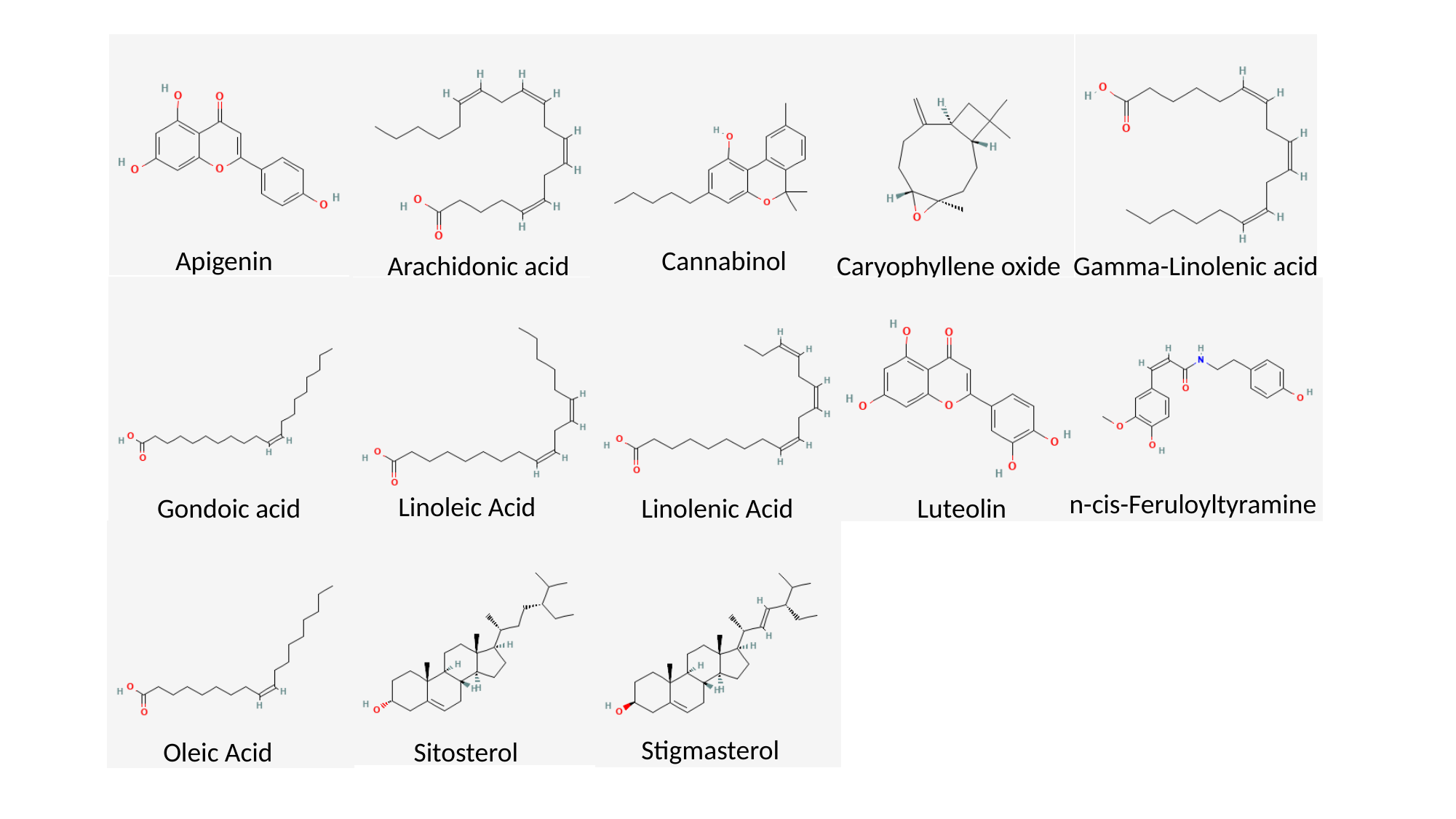

Apigenin
Cannabinol
Arachidonic acid
Caryophyllene oxide
Gamma-Linolenic acid
n-cis-Feruloyltyramine
Linoleic Acid
Gondoic acid
Linolenic Acid
Luteolin
Stigmasterol
Oleic Acid
Sitosterol
